# Atraumatic Bilateral Acute Compartment Syndrome of the Lower Legs: A Review of the Literature

**DOI:** 10.7759/cureus.20256

**Published:** 2021-12-08

**Authors:** Madeline Warren, Govind Dhillon, Joseph Muscat, Ali Abdulkarim

**Affiliations:** 1 Trauma and Orthopaedics, East and North Hertfordshire NHS Trust, Stevenage, GBR; 2 Trauma and Orthopaedics, University Hospitals Sussex NHS Foundation Trust, Brighton, GBR

**Keywords:** acute, rhabdomyolysis, spontaneous, atraumatic, bilateral compartment syndrome

## Abstract

Bilateral acute compartment syndrome of the legs is a very rare presentation that requires emergency surgical intervention. Atraumatic bilateral cases are almost unheard of in medicine. There is currently no link between compartment syndrome and cognitive impairment or mental health.

A systematic literature search was performed in accordance with the Preferred Reporting Items for Systematic reviews and Meta-Analyses guidelines using the following keywords in multiple databases: compartment syndrome, atraumatic, spontaneous, bilateral, both, lower leg, acute, compartmental pressure, and fasciotomy. Atraumatic, bilateral, acute, and confirmed compartment syndrome cases were included.

In total, 33 cases of atraumatic bilateral acute compartment syndrome (ABACS) were identified, of those 72.7% of cases were males. A form of cognitive impairment was found in 66% of cases. The medical history of the cases included substance abuse (nine patients), mental health illness (seven patients), and hypothyroidism (four patients). Within the reports, there was evidence of a misdiagnosis or delayed management in 19 cases (57.6%). Creatinine kinase (CK) was measured in 28 cases with a mean CK of 110,893 IU/L. Compartment pressure measurements were used in only 12 cases. A total of 29 cases were managed with bilateral four-compartment fasciotomy.

This review highlights that ABACS is a condition with high rates of misdiagnosis or delay in treatment. Associations found included patients with cognitive impairment on presentation, mental health conditions, substance misuse, and elevated levels of CK. In addition, this review demonstrates that this condition is less rare than previously thought with serious morbidity and mortality.

## Introduction and background

Acute compartment syndrome is an orthopaedic emergency. If left untreated, it leads to limb ischemia and potentially devastating sequelae including permanent nerve damage and disability, amputation, and death [1,2]. The most common causes of acute compartment syndrome are fractures (open/closed), soft tissue injury, rhabdomyolysis, and vascular impairment. Less commonly, burns, immobility, and infectious myositis can be causes [1,3]. Acute bilateral cases of compartment syndrome are a rare presentation outside the context of trauma or extreme exercise. They can be diagnostically challenging and may be under-recognised, leading to delayed diagnosis and poor outcomes [2].

Compartment syndrome occurs as a result of increased intra-compartmental pressure (ICP) impairing blood circulation to the limb. Blood circulation from high-pressure arteries to low-pressure veins depends upon the arteriovenous (AV) pressure gradient [2]. Increasing the ICP reduces the AV gradient and initially causes a reduction in the drainage of deoxygenated venous blood [2]. This reduction leads to the third spacing of the fluid, further increasing the ICP, resulting in a vicious cycle being established. Continuous increase in the ICP impairs the supply of oxygenated arterial blood to the limb, leading to irreversible ischaemia and necrosis of the muscles and nerves [2].

Diagnosis of compartment syndrome is often clinical, relying on the recognition of pain out of proportion to an apparent injury. Later signs such as paraesthesia, paralysis, or pulselessness typically occur at a stage of irreversible damage [2]. The pain associated with compartment syndrome is severe, out of proportion to the injury, and not relieved by analgesia or loosening of tight casts. On clinical examination, the compartments are often swollen and tense. The pain can be exacerbated by passively stretching the muscles within the compartments. However, these clinical signs require patients to verbalise symptoms which may not be possible in severely unwell or intubated patients [4]. In addition to clinical examination, measuring ICP can aid diagnosis. Normal compartment pressures range from 0 to 8 mmHg, with compartment pressures greater than 30 mmHg being indicative of compartment syndrome and warranting surgical intervention [2].

Despite recognition that compartment syndrome can occur in the absence of trauma, there is limited literature on the topic and even less data exist for acute bilateral atraumatic compartment syndrome (ABACS). In this literature review, we identify some of the main factors associated with this condition to establish some key learning points to aid diagnosis and prompt management for clinicians presented with such cases [5].

## Review

Methodology

This review was conducted in accordance with the Preferred Reporting Items for Systematic Reviews and Meta-analyses (PRISMA) guidelines where possible [6]. We systematically searched Medline/PubMed, EMBASE-Ovid, and Cochrane databases using MeSH and keywords with synonyms, as well as combinations of these terms. Our search included a combination of the following keywords: compartment syndrome, atraumatic, spontaneous, bilateral, both, lower leg, acute, compartmental pressure, and fasciotomy. We aimed to identify case reports or case series of patients who have had an episode of acute compartment syndrome that was atraumatic, in both lower legs, and diagnosed via fasciotomy, imaging, or pressure monitoring. Only English-language papers were included in this review.

Studies that were deemed eligible for inclusion met the following criteria: (1) bilateral compartment syndrome, (2) atraumatic, (3) acute compartment syndrome, (4) both legs in the same episode, and (5) English-language abstract available. In this study, trauma was defined as a direct external injury to the affected limbs and was considered absent if there was no clinical sign of soft tissue damage or fracture. The following studies were excluded: (1) cadaveric or animal studies, (2) compartment syndrome with preceding trauma or exercise (e.g., rigorous running), (3) unilateral cases, (4) classification studies, (5) morphology studies, (6) simulation studies, and (7) English-language abstract or paper unavailable.

Two authors (MW and GD) independently screened titles and abstracts using the Covidence software [7]. The full text was retrieved, reassessed against the above-mentioned inclusion and exclusion criteria, and any disagreements were resolved by discussion.

All identified studies were case reports, with an intrinsically high risk of bias and limited scope for data analysis. Data collection identified cases that have not been collated before, all occurred independently of each other and were reported with different information available. Variables collected from each case included gender, age, symptoms, history, cause, investigation, management, morbidity, and mortality.

Due to the low incidence of this injury, all evidence was included. Descriptive analysis was performed to generate common descriptive statistics with values reported as total number (n) and percentages (%).

Results

**Figure 1:**
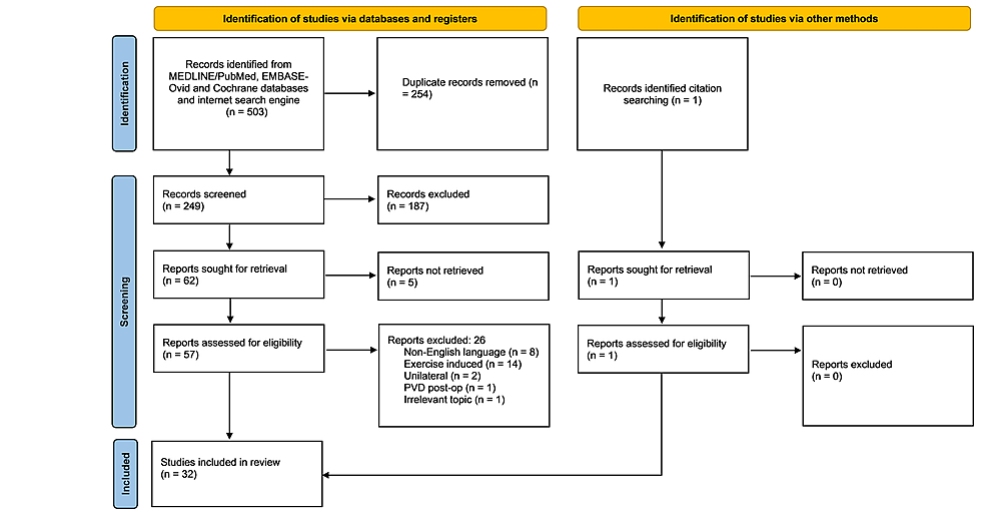
PRISMA 2020 flow diagram for ABACS review. PRISMA: Preferred Reporting Items for Systematic Reviews and Meta-Analyses; ABACS: acute bilateral atraumatic compartment syndrome

The search yielded 249 individual studies, of which 32 were identified as meeting the inclusion criteria. One study [8] was a case series that included two cases meeting the inclusion criteria, making the total 33 cases (Figure 1). The data extracted could not be put through any statistical analysis past summative calculations.

**Figure 2:**
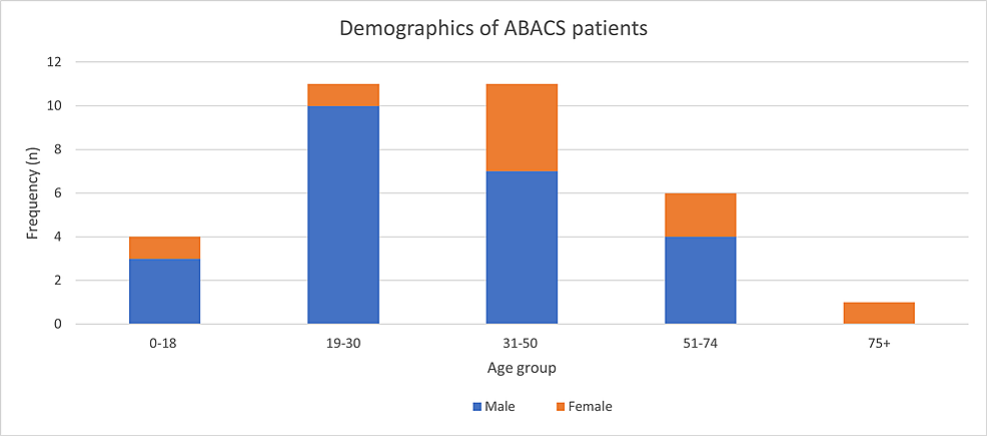
Bar chart showing cases by age group and gender. ABACS: acute bilateral atraumatic compartment syndrome

In total, 33 cases of ABACS were identified, including 24 (72.7%) males and nine (27.3%) females. There were 10 males aged 19-30 with ABACS, which was the largest sub-group. Among females, the age group with the highest frequency was 31-50 including four patients (Figure 2). The mean age of all patients in the study was 36.1 years (for males: the mean age was 32.7 years; for females, the mean age was higher at 45.1 years).

The most frequently occurring medical history of the cases included substance abuse (nine patients), mental health illness (seven patients), and hypothyroidism (four patients). A form of cognitive impairment was noted in 21 (63.6%) cases. Of those, six were under anaesthesia (at the time or recently), six were unconscious, six were intoxicated, and three had some form of mental disturbance.

**Figure 3:**
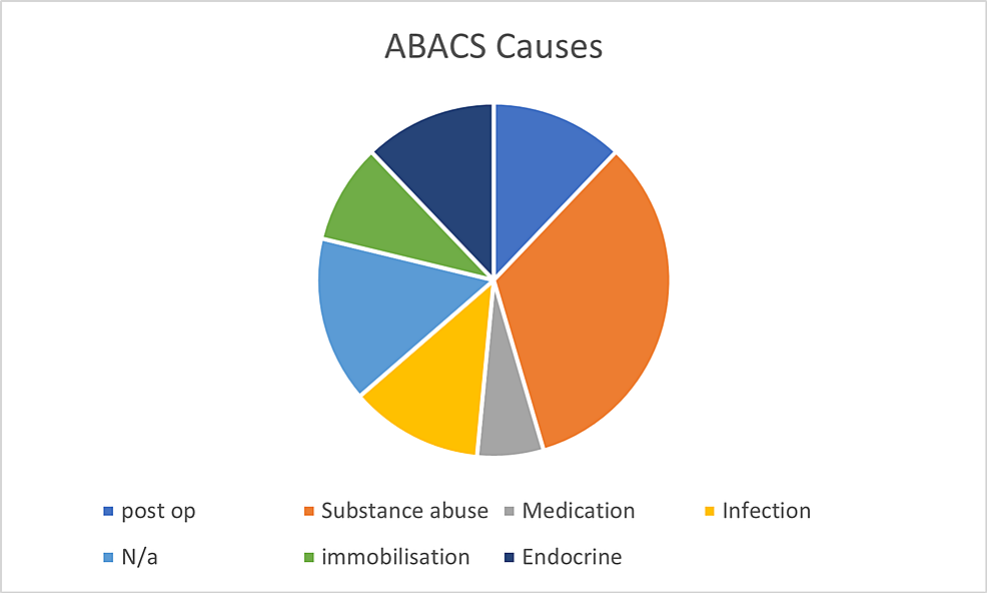
Pie chart showing the most likely cause of patients with ABACS. ABACS: acute bilateral atraumatic compartment syndrome; N/a = not applicable

The most likely causes for ABACS are presented in Figure 3, with the most common causes being related to substance abuse (33%), followed by post-operative, infection, and hormonal (serotonin syndrome, hypothyroid, and psychogenic polydipsia). No cause was identified or mentioned for five cases [9-13] (Figure 3). Within the reports, there was evidence of a misdiagnosis or delayed management in 19 (57.6%) cases (Table 1). Creatine kinase (CK) was measured in 28 cases, with the mean average CK of 110,893 (IU/L). Compartment pressure was used for diagnosis in 12 (36.4%) cases, with some studies reporting multiple sets of up to eight pressure readings at a time. Due to inconsistency in reporting compartment pressures, it was not possible to provide a representative mean pressure reading.

**Table 1:** Summary of notable results from the literature review. PMH: past medical history; CK: creatine kinase; Ix: investigations; Mx: management; M+M: morbidity and mortality; B/L: bilateral

	Yes	No	N/A	% Yes
PMH: Substance abuse	9	24	0	27.3%
PMH: Mental health	7	26	0	21.2%
PMH: Hypothyroid	4	29	0	12.1%
Presentation: Cognitive impairment	21	13	0	63.6
Ix: CK blood test used	28	0	5	84.8%
Ix: Compartment pressure used	12	0	21	36.4%
Ix: Delay/misdiagnosis	19	0	14	57.6%
Mx: B/L four-compartment fasciotomy operation	29	2	2	87.9%
Associated conditions/complications: Renal impairment	14	0	19	42.4%
Associated conditions/complications: Rhabdomyolysis	12	0	21	36.3%
Associated conditions/complications: Infection	11	0	22	33.3%
Complications: Post-operative wound infection	4	27	2	12.1%
M+M: B/L peroneal nerve palsy	10	5	18	30.3%
M+M: Full recovery	5	20	8	15.2%
M+M: Died	2	31	0	6.1%

Not all cases were isolated ABACS; one case had only bilateral anterior compartment syndrome [14], and another case had bilateral anterolateral compartment syndrome of the lower legs [13]. Other cases also had compartment syndrome in other areas of the body. Four cases underwent fasciotomy in one or more thighs as well as both lower legs [12,15-17]. Two other patients required fasciotomy of both forearms [18,19].

In total, 29 out of 33 cases were managed operatively with emergency bilateral fasciotomy. Only two cases were managed conservatively; one showed minimal improvement after stopping medication that was suspected to have caused it [20], and the other required a tibialis posterior transfer procedure to walk without orthotics [21]. Two cases did not contain sufficient information about management.

Associated conditions were reported in 29 cases, two had no other associated conditions [13,22], and two did not have information available [16,23]. The most frequently occurring complications or associated conditions were renal impairment (14 patients), rhabdomyolysis (12 patients), and infection (11 patients) (Table 2). Two patients required above the knee amputations; one patient had necrotizing fasciitis following a *Vibrio vulnificus* infection [24], and the other was after a prolonged kneeling [25]. Neither of the two cases reported a delay in diagnosis or management. Two patients died; one with delayed diagnosis after prolonged kneeling [26], and another without delay was likely due to drug abuse [8]. Table 2 summarises the individual studies and includes data that the authors of this review deemed relevant.

**Table 2:** A summary of cases included in the review with year, symptoms, history, cause, investigation, management, morbidity and mortality PMH: past medical history; IV: intravenous; CK = creatine kinase; b/l = bilateral; MDMA: 3,4-methyl​enedioxy​methamphetamine; PE: pulmonary embolism; IHD: ischaemic heart disease; AAA: abdominal aortic aneurysm; MRI: magnetic resonance imaging; UTI: urinary tract infection; GA: general anaesthesia; DVT: deep vein thrombosis; CKD: chronic kidney disease; HTN: hypertension; HIV: human immunodeficiency syndrome; AKI: acute kidney injury; ED: Emergency Department; DM: diabetes mellitus; NV: neurovascular; AKA: above-the-knee amputations; IVDU: intravenous drug users; IVC: inferior vena cava

Paper	Patient	PMH	History	Cognitive impairment	Symptoms/Signs	Suspected cause	CK (IU/L)	Urine dip	Use of compartment pressure?	Delay in diagnosis/Misdiagnosis	Management	Associated conditions	Morbidity/Mortality
Abdullah et al. (2006) [27]	30 M	Depression, cocaine abuse	Severe pain in the lower limbs after injecting IV heroin the day before	Yes – recent heroin use	Erythema, B/L weakness	Heroin overdose/positional	236,000	Yes – raised myoglobin	n/a	None	Operative	Rhabdomyolysis	Mild improvement in lower limb strength
Abrahamsen and Stilling (2013) [25]	39 F	Obese, opioid abuse, EtOH excess	Kneeling for 8 hours	Yes	Erythema	Positional	>20,000	n/a	n/a	No – symptoms after presentation	Operative	Renal failure, toxic shock, seizures	Transfemoral B/L amputation
Armstrong et al. (2019) [8]	29 M	MDMA use	Drug use, in circulatory shock, seizures	Yes – seizures and agitation at presentation	n/a	Synthetic cannabis	236,736	n/a	n/a	Yes – transferred to another hospital	Operative	Renal failure, liver failure	n/a
Armstrong et al. (2019) [8]	28 M	n/a	Drug use, aggressive, rolling in the street	Yes – agitated and needed intubation	n/a	Synthetic cannabis + cocaine	>320,000	n/a	n/a	n/a	Operative	Hepatic failure, lactic acidosis	Died
Ballesteros et al. (2008) [28]	49 M	Klippel-Feil syndrome, L4-S1 fusion	10-hour operation for spinal revision surgery kneeling position with thigh-high stockings. Return to theatre for misplaced L3 screw	Yes – under general anaesthetic	Swelling, tense legs	Positional + stockings + post-operative	n/a	n/a	Yes	Yes – taken back to theatre for misplaced screw	Operative	Rhabdomyolysis	n/a
Biswas et al. (2013) [29]	22 F	Asthma, bipolar, hypothyroid, marijuana/alcohol misuse	2 days kneeling in a closet after IV heroin OD, found unconscious	Yes – unconscious	Swelling, tender, petechiae	Positional secondary to heroin use	160,000	Yes – blood	Yes	No	Operative	Rhabdomyolysis, renal failure, gastritis	n/a
Blanchard et al. (2013) [26]	75 F	PE, IHD, high cholesterol, AAA, sciatica	Kneeling and unable to get up for 4 hours	No	Swelling, reduced sensation, reduced power plantar/dorsiflexion, tense	Positional	40,517	Yes – nitrates, leukocytes, white cells	Yes	Yes – waited for MRI imaging before theatre	Operative	Rhabdomyolysis, UTI, upper limb ischaemia	Died on day 2
Chin et al. (2009) [30]	44 F	Ulcerative colitis	Lithotomy position for 7 hours, proctectomy operation	Yes – post-operative GA	Left-sided absent pulses	Positional + post-operative	35,000	n/a	n/a	Yes – given heparin for suspected DVT	Operative	Rhabdomyolysis	B/L footdrop requiring splints and walking aid at 2 months post-operatively
Chaudhary et al. (2015) [20]	51 M	Hypothyroid, high cholesterol	1 week after starting thyroxine and statin	No	Tense, swelling, erythema, weak dorsiflexion, b/a football	Drug-induced, hypothyroid	6,459	n/a	n/a	Yes	Conservative – stopping statin	Hypothyroidism with severe myopathy and rhabdomyolysis	Minimal improvement after stopping medication
Clarissa Samara and Warner (2017) [31]	68 F	CKD, HTN, depression	Developed encephalopathy, found unconscious, on paroxetine, risperidone, and dextroamphetamine	Yes - unconscious	Tense, hyperreflexia, clonus	Serotonin syndrome	84,166	n/a	n/a	No – developed symptoms on day 3 of presentation	Operative	Serotonin syndrome, sepsis	Unable to ambulate 6 weeks post-operatively
Davidson et al. (2013) [10]	31 M	HIV, hepatitis B	Woke with leg pain, mild yoga the night before	No	Pain on passive stretch, reduced sensation, dark urine	No cause	200,323	n/a	Yes	Yes – diagnosed initially with vascular pathology and transferred to vascular unit	Operative	AKI	Decreased sensation in dorsum foot B/L
Doddi et al. (2009) [32]	40 M	None	1 day of pain in legs, vomiting	None	Swelling, tense, pain, passive flex	Infection with *S. pneumoniae*	n/a	Yes – NAD	n/a	n/a	Operative	AKI, oliguric, sepsis	None
Figueras Coll et al. (2015) [15]	49 M	Opioid addiction	Intoxicated on alcohol, leg and thigh pain	Yes – intoxicated	Tachycardic, tender, no pulses,	Alcohol	66,000	n/a	Yes	Yes – sent home from ED day before	Operative (B/L lower leg and thigh fasciotomy)	Thigh B/L compartment, hyperkalaemia, acidosis	None
Godeiro-Júnioret al. (2007) [23]	25 M	Gynecomastia, anabolic steroid use, amphetamine use	Rhytidoplasty (40 minutes) procedure under GA, agitated post-operatively, pain in the legs	Yes – post GA and agitated post-operatively	B/L oedema, painful dorsiflexion, weakness dorsiflexion	Steroid use	4,3240	no	n/a	Yes – nuroleptic malignant syndrome	Operative		B/L peroneal nerve palsy
Goldin et al. (2010) [33]	58 F	Obese	12 hours sitting in front of a slot machine and binge drinking	Yes – intoxicated	Paraparesis, B/L peroneal nerve palsy	Positional/Alcohol	n/a	n/a	n/a	n/a	n/a	Rhabdomyolysis, sepsis, multiorgan failure	B/L peroneal nerve palsy, walks with brace B/L
Goru and Goru (2012) [34]	41 F	Alcohol abuse	Calf pain for 5 hours, passing dark urine	Yes – intoxicated	Blisters, absent PT pulse, delayed capillary refill	Alcohol	37,400	No	n/a	Yes – management for allergic reaction	Operative	Renal failure, hyperkalaemia	n/a
Kapur et al. (2015) [11]	58 M	Schizophrenia	In care home for 2 days, reduced mobility	Yes – Schizophrenia	B/L leg pain, swelling, erythema	n/a	6,966	n/a	n/a	Yes – initially treated for cellulitis	n/a	AKI, rhabdomyolysis, small bowel obstructions	n/a
Kasugai et al. (2020) [18]	18 F	Depression, anorexia, suicidal attempts	Overdose on diabetic medications, unconscious on arrival	Yes – unconscious	Legs tense, oedema developed on day 4	Positional/Overdose of DM medications	184,200 (day 4)	n/a	Yes	Yes – initially diagnosed with rhabdomyolysis only	Operative (B/L lower leg and forearm)	B/L forearm compartment syndrome	B/L peroneal nerve palsy
Khan et al. (2012) [9]	41 F	None	Difficulty walking, passing dark urine	No	B/L reduced sensation, tense	n/a	374,800	n/a	n/a	Yes – initially treated for allergy	Operative	Renal failure, rhabdomyolysis	Necrosis to muscle, no NV deficit
Lu et al. (2008) [24]	29 M	Renal failure on regular dialysis	1-day fever, cough, left calf pain	No	Warm, swelling, tender, firm, pain on passive movements	Necrotizing fasciitis from *V. vulnificus*	1,401	n/a	n/a	No	Operative (left leg D0 right leg D3)	Sepsis, necrotizing fasciitis	D4 required left leg AKA
Luzzi et al. (2008) [35]	2 M	Abdominal mass	4 hours post right thoracotomy for abdominal neuroblastoma, post-operative leg and forearm pain, hypocalcaemia	Yes – child and post GA	Dorsiflexed limbs bilaterally	Hypocalcaemia/post-operatively	n/a	n/a	Yes	Yes – orthopaedic review on day 4 post-operatively	Operative	Hypocalcaemia	No morbidity at the 5-year follow-up
Maiocchi and Bernardi (2012) [14]	31 M	G6PD, schizophrenia, drug abuse, psychogenic polydipsia, hyponatremia, seizures	Water intoxication led to hyponatraemia, in recovery patient developed rhabdomyolysis and B/L pain in the legs	Yes – schizophrenic	Erythema, warm	Psychogenic polydipsia	10,800	Yes – raised myoglobin	Yes	Yes – diagnosed correctly on the third presentation	Operative – anterior compartments only	Hyponatraemia	B/L foot drop requiring long-term orthotics
Muir et al. (2012) [21]	22 M	Shin splints (1/12)	1-month history of fatigue and shin splints, 3 days of altered sensation in the feet, swelling	None	Hyperpigmentation, reduced power dorsiflexion	Primary adrenal insufficiency/hypothyroid	>25,000	n/a	n/a	n/a	Conservative	Rhabdomyolysis, renal failure, hypothyroid, hyperkalaemia	Required tibialis posterior transfer to walk without orthotics
Ng et al. (2008) [19]	18 M	Allergic rhinitis, chronic otitis media	Self-presented with cough, myalgia, ear discharge, limb pain, deteriorated, intubated	Yes – intubated	Paraesthesia, swelling, weak dorsiflexion	Infection/Sepsis	538,000	Yes – myoglobin	Yes	Yes – CT scan looking for nerve entrapment	Operative	Left forearm compartment, renal failure	8 months weak dorsiflexion requiring foot orthosis
Ochoa-Gómez et al. (2002) [36]	35 M	IVDU, HIV, hepatitis C	Found unconscious in a prison cell, septic shock, cardiac arrest, developed leg symptoms 36 hours after admission, drug screen positive for opioids, cannabis	Yes – unconscious	Oedema, lack of peripheral pulses	Infection, drug overdose	28,750	n/a	n/a	n/a	Operative aponeurotomy	Septic shock, cardiac arrest	n/a
Paletta et al. (1993) [17]	6 M	none	3 weeks fever and sore throat, oedema of legs		Tense, tender, reduced pulses	Myositis from influenza	303,200	n/a	Yes	Yes – 12 hours	Operative	B/L thigh compartment syndrome, renal failure	Normal function at 12 months
Parzych et al. (2019) [16]	37 M	n/a	Heroin overdose, asleep for long hours (unknown), resulted in leg and thigh compartment	Yes – unconscious	n/a	Drugs, positional	n/a	n/a	n/a	n/a	Operative	n/a	n/a
Ramdass et al. (2007) [22]	54 M	Hypothyroid, high cholesterol	Recently admitted for Graves’ disease, recently started on simvastatin	No	Tense, swelling	Simvastatin	6,000	n/a	n/a	No	Operative	None	n/a
Shokoohi et al. (2008) [12]	54 M	Globe melanoma, PE, IVC filter,	Acute-onset abdominal and back pain, B/L leg pain	Yes – agitated	Oedema, reduced power and sensation	n/a	7,988 (day 2)	n/a	Yes	Yes – 18 hours for diagnosis	Operative	Renal failure, rhabdomyolysis, gangrene, thigh B/L compartment	n/a
Smith et al. (1998) [13]	24 M	T1DM, hypothyroid, smoker	4 days of shin pain, reduced sensation, feverish	None	Warm, tense, swollen, weak dorsi/plantarflexion, reduced sensation	n/a	n/a	n/a	Yes	No	Operative (anterolateral B/L fasciotomy)	None	Tendon transfer for B/L footdrop, reduced sensation foot
Sofat et al. (1999) [37]	25 M	None	42-unit alcohol binge, slept on the floor for 12 hours, after 2/7 history of leg pain, swelling, anuria, dark urine from catheter, in shock	No	B/L footdrop, absent reflexes, reduced sensation	Alcohol binge, positional	>10,000	Yes – blood/protein	n/a	Yes – 6 hours until operation	Operative	Hyperkalaemia, renal failure, rhabdomyolysis	Could walk unaided after 5 months
Tuckey et al. (1996) [38]	28 M	Ulcerative colitis	8-hour procedure for ileal pouch formation procedure under GA and epidural in lithotomy position with thigh high stockings, developed pain 15 hours post-operatively	Yes – recent GA	Tense, swollen legs, pain on passive dorsiflexion	Positional lithotomy position and stockings	n/a	n/a	n/a	No – immediately back to theatre	Operative	Wound infection – S. aureus, septicaemia	Long-term B/L foot drop and needed foot orthoses for life
Ulstrup et al. (2015) [39]	30 M	Anxiety, delusions, schizophrenia	Found unconscious in a psych hospital, on aripiprazole, water intoxication, regained consciousness.	Yes unconscious and schizophrenia	Erythema, tense, weak dorsiflexion	Psychogenic polydipsia	29,900	n/a	n/a	Yes – ortho review on day 3 and suspected necrotising fasciitis	Operative	Sepsis	Needed long-term orthotics

Discussion

To the authors’ knowledge, this is the largest literature review of ABACS to date. A previous study from 2012 identified eight cases published between 1993 and 2009 [9]. Our study identified 15 cases from the same time frame, along with 17 reported in the 12 years since. There are undoubtedly unpublished cases, making this condition more prevalent than previously thought [9]. While still rare, ABACS should be considered a recognised phenomenon and always be included in the differential diagnosis for patients presenting with bilateral leg pain, even in the absence of trauma. Our study has also identified correlated features which can hopefully expedite the diagnosis of this limb-threatening condition.

The most striking finding from our study is the high association with a history of mental health conditions and cognitive impairment. This may be due to the clinical features of the conditions, side effects of medication, or higher rates of substance abuse and associated behavioural factors.

Two patients in our review with known mental health disorders suffered from schizophrenia, which can be associated with hyposensitivity to pain. The cause of this is unknown but has previously been noted in an atypical presentation of compartment syndrome following a tibial fracture [40,41]. Rhabdomyolysis has been recognised as a rare complication of several anti-psychotic medications and is known to be associated with compartment syndrome, with some reporting it in up to 23% of cases [42,43].

The added complication of cognitive impairment makes diagnosis extremely difficult and likely contributes to the high rate of delayed diagnosis in this series. In trauma management, we recognise the difficulty of diagnosing compartment syndrome in moribund patients and have a high index of suspicion because of low thresholds for compartment pressure monitoring [44]. Similarly, a high level of clinical vigilance and continuous monitoring is needed in those with impaired cognitive function if patients are unable to fully cooperate with a clinical examination or clearly articulate their symptoms [45].

Multiple cases in our review highlight the association between intoxication from both alcohol and drug use, resulting in a prolonged state of immobilisation, and the subsequent presentation of compartment syndrome complicated by rhabdomyolysis. Acute alcohol intoxication with prolonged immobilisation is one of the most common causes of rhabdomyolysis [46], and abnormal CK levels are present in almost half of those who attend the emergency department following recreational drug use [47].

Other causes included hypothyroidism [20,21] and rhabdomyolysis secondary to influenza infection [17,19]. The aetiology of hypothyroidism-induced compartment syndrome is not clear, although theories involving the alteration of the synthesis of glycosaminoglycans have been suggested [48]. Direct viral infection of the muscle with influenza causes myositis, resulting in muscle oedema and swelling and subsequent compartment syndrome [49].

Over half of the patients in this review (57.6%) had a delayed or missed diagnosis. A delay of 6-120 hours is known to be associated with high rates of amputation [50,51]. The most common complication from delayed diagnosis in our review was a bilateral peroneal nerve palsy, with 12 (36.4%) patients having varying degrees of long-term impairment ranging from numbness and foot drop and two patients requiring a tendon transfer to improve mobility. Delay in the treatment of compartment syndrome worsens the severity of nerve injury [52].

Measurement of CK may aid in the diagnosis, although it is not currently recommended in the United Kingdom [53]. One study using CK as an adjunctive marker found that levels greater than 4,000 U/L are suggestive of the condition. CK levels in our study were 10 times higher on average, which may be attributed to more extensive damage demonstrated by bilateral symptoms or could be related to increased muscle death from delayed diagnosis [54].

Diagnosing compartment syndrome using clinical findings is challenging as the findings have low sensitivity and positive predictive value and their absence may be more useful to exclude the diagnosis [55]. Among clinicians, there is a wide variation on what constitutes a diagnosis of compartment syndrome based on clinical signs, which can lead to unnecessary operations or potential delays to theatre [56]. In our review, measurement of compartment pressures occurred in 12 (36.4%) cases. Although there are potentially numerous limitations to compartment pressure monitoring [57], many have shown ICP monitoring to be accurate, sensitive, and reliable with various available techniques [44,58]. In cases of diagnostic uncertainty or where the patient is unable to cooperate with the examination, ICP monitoring is important to avoid a delay in diagnosis [44,59].

The greatest limitation of this review is that it was limited to case studies of ABACS; however, we believe this represents the highest level of evidence currently available for this rare presentation. Associations with cognitive impairment and alcohol or drug intoxication need to be clarified with larger studies but may aid clinicians in recognising this life-changing condition. Future research could also assess whether greater use of compartment pressure monitoring and early measurement of CK improves time to diagnosis or reduces morbidity.

## Conclusions

ABACS should be considered a recognised phenomenon and always included in the differential diagnosis for patients presenting with bilateral leg pain, even in the absence of trauma. The cases identified were predominantly males approximately 30 years of age. There was also a high prevalence of cognitive impairment in published cases for this condition and many had a background of mental health conditions. Typical presentations include those who have taken or are under the influence of alcohol or other drugs, where a period of a long lie secondary to a comatose state has occurred.

CK is often elevated and can be a useful adjunct in helping to make the diagnosis in case of doubt, although this is not currently part of UK guidelines. More extensive use of ICP monitoring may help reduce delay in diagnosis and treatment in cases where clinical signs are equivocal. Clinicians should be wary about the potential complication of rhabdomyolysis with subsequent renal impairment and should monitor these patients closely. More than half of the patients with ABACS have a delayed diagnosis or misdiagnosis for a condition with high morbidity and mortality; this paper aims to improve the diagnosis moving forward.
